# Immunogenicity and reactogenicity of repeated intradermal mRNA COVID-19 vaccines administered as a second booster dose in a Thai geriatric population

**DOI:** 10.3389/fimmu.2023.1302041

**Published:** 2024-01-11

**Authors:** Prasert Assantachai, Suvimol Niyomnaitham, Zheng Quan Toh, Monthira Thammasalee, Napaporn Pengsorn, Wiyachatr Monklang, Paul V. Licciardi, Kulkanya Chokephaibulkit

**Affiliations:** ^1^ Department of Preventive and Social Medicine, Faculty of Medicine Siriraj Hospital, Mahidol University, Bangkok, Thailand; ^2^ Department of Pharmacology, Faculty of Medicine Siriraj Hospital, Mahidol University, Bangkok, Thailand; ^3^ Siriraj Institute of Clinical Research (SICRES), Faculty of Medicine Siriraj Hospital, Mahidol University, Bangkok, Thailand; ^4^ Infection, Immunity and Global Health, Murdoch Children’s Research Institute, Parkville, VIC, Australia; ^5^ Department of Pediatrics, The University of Melbourne, Parkville, VIC, Australia; ^6^ Department of Pediatrics, Faculty of Medicine Siriraj Hospital, Mahidol University, Bangkok, Thailand

**Keywords:** repeated dose, intradermal vaccination, fractional dose, intramuscular vaccination, COVID-19, second booster, older adult

## Abstract

**Background:**

Geriatric populations are at an increased risk of severe presentations, hospitalization, and loss of life from COVID-19. Few studies have explored vaccination regimens in adults >65 years old. Repeated booster vaccination is required for high-risk populations as COVID-19 vaccine efficacy is short-lived. We compared the immunogenicity and reactogenicity of second intradermal (ID) COVID-19 booster vaccination with second intramuscular (IM) vaccination in older adults.

**Methods:**

This single-center, open-labeled, prospective, cohort study conducted at Siriraj Hospital enrolled older adults ≥65 years old who previously received a first booster (third dose) mRNA vaccine (mRNA-1273 or BNT162b2) via ID or IM administration. Participants were allocated to receive a second booster of the same vaccine type and route as their first booster 16–17 weeks thereafter. Anti-SARS-CoV-2 receptor binding domain IgG and neutralizing antibody titers against Wuhan and Omicron subvariants (BA.1, BA.2, and BA.4/5) were measured 2 weeks after vaccination.

**Results:**

Of 91 enrolled participants, 72.5% were women, with a median age of 75 years. Forty-nine participants (53.8%) received a second ID booster, and 42 (46.2%) received a second IM booster. Two weeks after the second booster, all groups generated anamnestic IgG antibody responses that were 5.41- to 10.00-fold higher than at baseline. Overall, higher antibody GMTs against Wuhan and Omicron subvariants were observed in IM compared with ID regimens. ID mRNA-1273 induced similar GMTs to IM BNT162b2 2 weeks after the second booster against Wuhan (486.77 [321.48, 737.05] vs. 472.63 [291.24, 767.01], respectively; *p* = 0.072). Higher GMTs against Omicron BA.1 (GMR [95% CI], 1.71 [1.39, 2.11]; *p* = 0.023), BA.2 (1.34 [1.11, 1.62]; *p* = 0.845), and BA.4/5 (1.10 [0.92, 1.33]; *p* = 0.531) were seen in all groups at 2 weeks after the second booster compared with 2–4 weeks after the first booster. Both local and systemic AEs were less frequent after the second than after the first booster, regardless of administrative route and vaccine type. Local AEs were significantly more frequent in ID mRNA-1273 arms than their respective BNT162b2 arms 2 weeks after the second booster (ID-mRNA-1273 vs. ID-BNT162b2: *p* ≤ 0.001).

**Conclusion:**

Repeated fractional ID vaccination may be an alternative booster vaccination strategy for geriatric populations.

## 1 Introduction

A second COVID-19 booster (fourth dose) has been recommended for geriatric, and other high-risk, populations due to their increased risk of developing severe COVID-19, hospitalization, and/or death (1). Several studies in healthcare workers demonstrated that a second booster is safe, immunogenic, and had moderate efficacy against symptomatic infection (2–4). One study in adults aged ≥60 years found that a second booster vaccine was safe and reduced hospitalization by 64% (5).

Intradermal (ID) vaccination with dose-sparing approaches helps alleviate limited access to vaccine supplies, particularly in low- and middle-income countries (6). We previously found that the administration of a reduced dose (fractional) mRNA vaccination as a primary series (7) or first booster (third dose) (8, 9) was both safe and immunogenic in adults. In particular, fractional ID mRNA-1273 booster doses in older adults (≥65 years) induced humoral and cellular immune responses that were not significantly different from a standard intramuscular (IM) BNT162b2 booster (9). This suggests that ID vaccination may be an alternative option for next-generation variant vaccines, settings with limited vaccine supplies, or settings with multiple available vaccine types. In addition, despite the tendency for higher local AEs in ID than IM vaccination routes (particularly for mRNA-1273), fewer systemic AEs were observed in ID than IM mRNA-1273 and BNT162b2 booster vaccine regimens (9).

Few studies explored these vaccination regimens in older adults (>65 years). The available immunogenicity data of repeated ID vaccination is also limited. AEs after a second booster are also a concern. This study assessed the immunogenicity and reactogenicity of repeated fractional ID vaccination as a second booster.

## 2 Materials and methods

### 2.1 Study design and participants

This single-center, open-labeled, prospective, cohort study was conducted at Siriraj Hospital, Bangkok, Thailand from 9 January to 8 August 2022. ≥65-year-old participants who received ID or IM mRNA-1273 or BNT162b2 as a first booster 16–17 weeks prior in our previous study (9) were eligible for enrollment. All participants previously received two doses of IM ChAdOx1 as their primary series. Those with a history of SARS-CoV-2 infection, those with acute illness or inflammation, those with a history of anaphylaxis to any vaccines or drugs, those who received any vaccination within 2 weeks of the study, those on immunosuppressive treatments, or those who were immunosuppressed were excluded. This study was registered under the Thai Clinical Trials Registry (TCTR20220112002), approved by the Siriraj Institutional Review Board (COA no. Si 335/2022), and carried out according to the International Council on Harmonization’s Good Clinical Practice (13th Edition), Belmont Report, and Declaration of Helsinki. All participants provided written informed consent before enrollment.

### 2.2 Study procedure

Participants were allocated to receive the same vaccine type and route as their first booster: 20 µg of ID-mRNA-1273 (0.10 mL), 50 µg of IM-mRNA-1273 (0.25 mL), 10 µg of ID-BNT162b2 (0.10 mL), and 30 µg of IM-BNT162b2 (0.30 mL). The different dosages between the first (100 µg, 0.50 mL) and second (50 µg, 0.25 mL) IM-mRNA-1273 boosters were because of modified recommendations by the Thai Ministry of Public Health following the recent approval of 50 µg of mRNA-1273 by the Thai Food and Drug Administration as booster vaccines in Thailand.

ID vaccinations were administered in the deltoid muscle region using the Mantoux technique. Wheals were measured to verify correct injection technique [ ≤ 4–8 mm in diameter, as described previously (9)].

### 2.3 Measured outcomes

Blood samples were collected before (baseline) and 2 weeks after the second booster for anti-SARS-CoV-2 receptor binding domain (RBD) IgG and 50% pseudovirus neutralizing titers (PVNT_50_) against the ancestral (Wuhan) strain and Omicron subvariants (BA.1, BA.2, BA.4/5). Anti-RBD IgG against SARS-CoV-2 spike protein (S1 subunit) were determined through chemiluminescent microparticle immunoassays (CMIA) using the SARS-CoV-2 IgG II Quant (Abbott, List No. 06S60) on the ARCHITECT I System. The assay measured antibody levels between 21.0 and 40,000.0 in arbitrary units (AU)/mL, later converted to the World Health Organization’s International Standard concentration of binding antibody unit per mL (BAU/mL) through the equation BAU/mL = 0.142 AU/mL provided by the manufacturer. Pseudovirus neutralization assays (PVNT) were performed as previously described (10). PVNT_50_ was defined as the highest serum dilution that reduced virus infectivity by 50% relative to control wells with no serum. The minimum detection limit was 1:40. Titers lower than this limit of detection (LOD) (<40) were assigned values of 20.

Participants were observed for at least 30 min after vaccination for immediate AEs. Participants and their caretakers were instructed to submit self-assessments of solicited local (i.e., pain, erythema, injection site swelling/induration, localized axillary lymphadenopathy, or swelling/tenderness ipsilateral to the injection arm) and systemic (i.e., headaches, fatigue, myalgia, arthralgia, diarrhea, dizziness, nausea/vomiting, rash, fever, and chills) AEs 7 days after vaccination using electronic diaries (Google Forms). Follow-up phone calls were performed by medical personnel 3–5 days after vaccination to verify self-reported AEs. Local and systemic AE severity were graded using a numerical rating scale of 1–4 based on the Common Terminology Criteria for Adverse Events (v5.0) guide published by the United States National Cancer Institute (NCI/NIH) (11).

### 2.4 Statistical analyses

AE endpoints were presented as frequencies and chi-square tests or Fisher’s exact tests used to assess statistical differences. Immunological endpoints were reported as geometric mean concentrations (GMCs) and geometric mean titers (GMTs) with 95% confidence intervals (CIs), respectively. Unpaired *t*-tests were used to compare IgG GMCs between groups. We compared immunogenicity and AE (second booster dose) data from this study with our previous study (first booster dose data) (9). All statistical analyses were performed using GraphPad™ Prism 9 (v9.2.0, 283; GraphPad™ Software, CA, USA) except for anti-RBD IgG comparisons between different age groups, which were performed using ANOVA through STATA (v17, StataCorp™, LP, College Station, TX, USA). *p* ≤ 0.05 was set as the statistically significant cutoff point.

## 3 Results

### 3.1 Baseline characteristics

**Figure 1:**
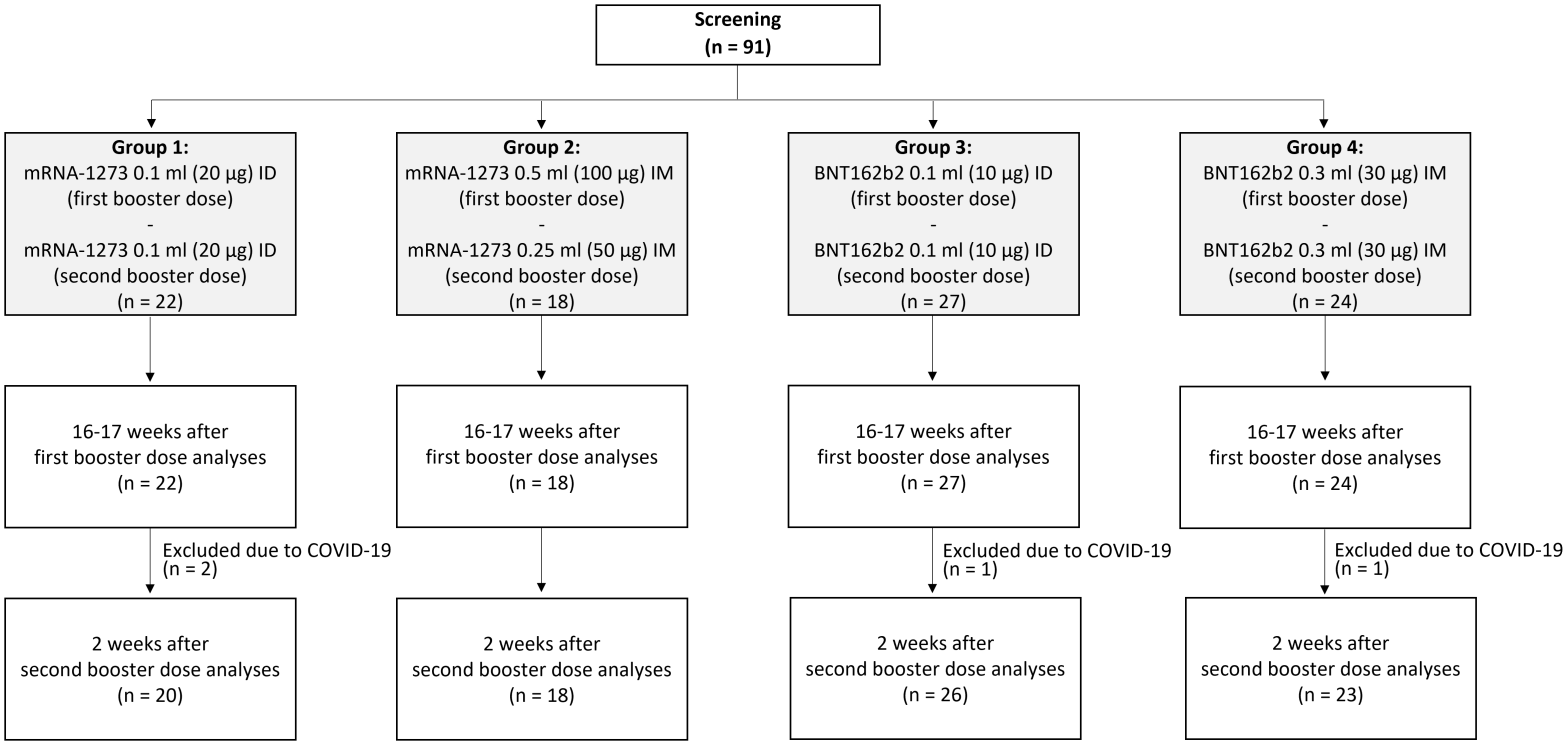
Consort flow diagram. A total of 91 participants were screened, and all deemed eligible for enrollment. Participants were allocated into four arms based on their first booster regimen. Participants were assessed before the administration of their second booster (16–17 weeks after their first booster) and at 2 weeks after receiving a second booster.

Of 91 enrolled participants, 72.5% (*n* = 66) were women, 49.5% (*n* = 45) had a normal body mass index (BMI), and the median (interquartile range) age was 75 (71–84) years (**Table 1**). Forty-nine (53.8%) participants received repeated ID boosters, while 42 (46.2%) received repeated IM boosters. The median time between the first and second booster was 17 weeks. Four participants developed SARS-CoV-2 infection and were excluded from the analyses (**Figure 1**).

**Table 1:** Baseline characteristics of participants included in the study.

First booster - Second booster	Vaccine Type					*p*-value
	All	mRNA-1273 20 µg ID - mRNA-1273 20 µg ID	mRNA-1273 100 µg IM - mRNA-1273 50 µg IM	BNT162b2 10 µg ID - BNT162b2 10 µg ID	BNT162b2 30 µg IM - BNT162b2 30 µg IM	
Number of subjects, *n* (%)	91 (100.00)	22 (24.18)	18 (19.78)	27 (29.67)	24 (26.37)	
Age (yrs, median [IQR])	75.00 (71.00, 84.00)	73.00 (71.00, 82.00)	77.50 (70.00, 83.00)	80.00 (72.00, 86.00)	74.00 (70.00, 82.00)	0.477
Female, *n* (%)	66 (72.53)	18 (81.82)	14 (77.78)	17 (62.96)	17 (70.83)	0.480
BMI (kg/m^2^, median [IQR])	24.40 (21.60, 26.80)	23.90 (20.20, 26.40)	25.85 (22.50, 28.20)	23.90 (21.40, 26.60)	24.25 (21.35, 27.30)	0.238
Underweight (BMI < 18.5 kg/m^2^, *n* [%])	4 (4.40)	1 (4.55)	0 (0.00)	2 (7.40)	1 (4.17)	0.653
Normal (BMI 18.5–24.9 kg/m^2^, *n* [%])	45 (49.45)	11 (50.00)	7 (38.89)	15 (55.56)	12 (50.00)	
Overweight (BMI ≥ 25.0 kg/m^2^, *n* [%])	42 (46.15)	10 (45.45)	11 (61.11)	10 (37.04)	11 (45.83)	
Interval between first and second booster (wks, median [IQR])	17.00 (16.00, 17.00)	17.00 (17.00, 18.00)	17.50 (17.00, 18.00)	16.00 (16.00, 17.00)	16.00 (16.00, 16.00)	0.002

IM, intramuscular injection; ID, intradermal injection; µg, micrograms; IQR, interquartile range; wks, weeks; BMI, body mass index; yrs, years.

### 3.2 SARS-CoV-2 anti-RBD IgG responses

**Figure 2:**
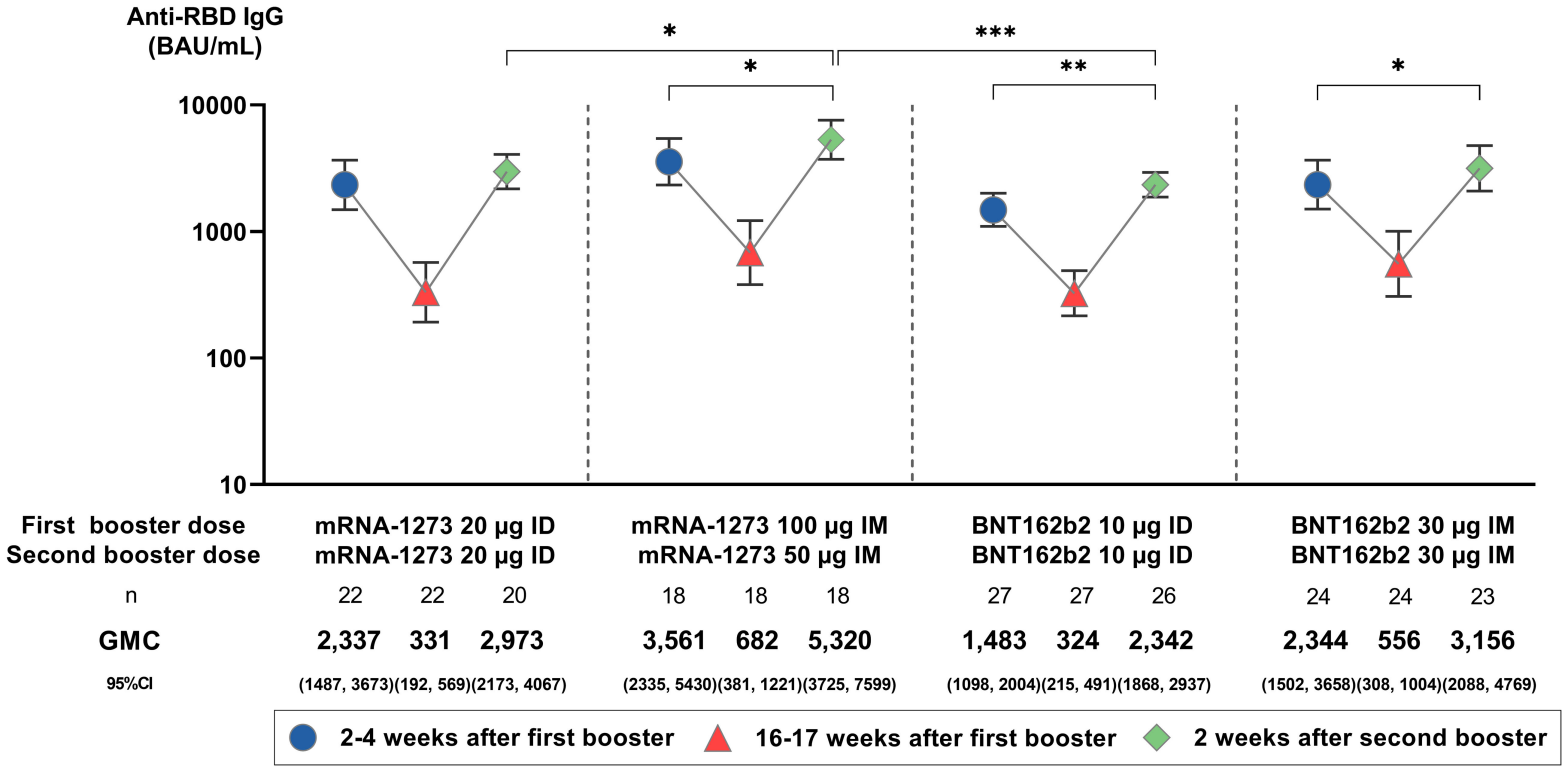
Anti-SARS-CoV-2 receptor binding domain (RBD) IgG geometric mean concentrations (GMC) before and after the administration of a second booster. GMCs are displayed with 95% confidence intervals (CIs) and were compared using unpaired Student’s *t*-tests. Only statistically significant *p*-values are displayed, with *, **, and *** denoting *p* ≤ 0.05, ≤ 0.01, and ≤ 0.001, respectively. µg, micrograms; BAU/mL, binding antibody units per mL; ID, intradermal; IM, intramuscular.

At 16–17 weeks after the first booster dose, anti-RBD IgG GMCs decreased across all groups by 4.22- to 7.07-fold. Two weeks after the second booster dose, 5.41- to 10.00-fold anamnestic responses from baseline (16–17 weeks after the first booster) were observed for all groups (*p* = 0.064; **Figure 2**). These titers were 1.38- to 1.57-fold higher than anti-RBD IgG levels generated 2–4 weeks after the first booster (*p* ≤ 0.05 for IM mRNA-1273 and BNT162b2, *p* ≤ 0.01 for ID BNT162b2). GMCs (95% CI) were highest in IM mRNA-1273 (5,320.38 BAU/mL [3,725.03, 7,598.98]), followed by IM BNT162b2 (3,115.56 BAU/mL [2,087.82, 4,769.35]), ID mRNA-1273 (2,972.77 BAU/mL [2,173.06, 4,066.77]), and ID BNT162b2 (2,341.98 BAU/mL [1,867.83, 2,936.50]) (*p* = 0.006; **Figure 2**; **Supplementary Table 1**). While anti-RBD IgG GMCs were higher in IM regimens than their respective ID regimens (*p* = 0.014 for IM vs. ID mRNA-1273, *p* = 0.183 for IM vs. ID BNT162b2), anti-RBD IgG GMCs induced by the ID mRNA-1273 regimen were not significantly different from the IM BNT162b2 regimen (**Figure 2**). This trend was consistent across all three time points.

### 3.3 NAb against SARS-CoV-2 Wuhan and Omicron subvariants

**Figure 3:**
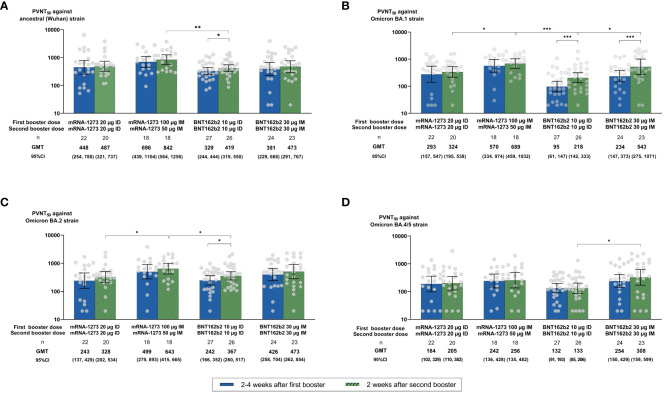
Neutralizing antibody (NAb) titers of 50% pseudovirus neutralization assays (PVNT_50_) against **(A)** the ancestral (Wuhan) strain and Omicron **(B)** BA.1, **(C)** BA.2, and **(D)** BA.4/5 subvariants at 2–4 weeks after the first booster and at 2 weeks after the second booster dose. Geometric mean titers (GMTs) are displayed with 95% confidence intervals (CIs) and were compared using unpaired Student *t*-tests. Only statistically significant *p*-values are displayed, with *, **, and *** denoting *p* ≤ 0.05, ≤ 0.01, and ≤ 0.001, respectively. µg, micrograms; ID, intradermal; IM, intramuscular.

Higher proportions of seropositive participants (PVNT_50_ ≥ 1:40) and GMTs against Omicron subvariants BA.1, BA.2, and BA.4/5 were observed in all groups at 2 weeks after the second booster compared with at 2–4 weeks after the first booster (95.4%, 97.7%, and 86.2% vs. 85.7%, 93.4%, and 83.5%, respectively; **Figures 3B–D**; **Supplementary Table 1**). No significant differences in seropositive rates against Omicron subvariants BA.1, BA.2, and BA.4/5 were seen across vaccine types (mRNA-1273 vs. BNT162b2: 97.4% vs. 93.9%, *p* = 0.629; 97.4% vs. 98.0%, *p* = 0.999; 86.8% vs. 85.7%, *p* = 0.880) or administration routes (ID vs. IM: 95.7% vs. 95.1, *p* = 0.999; 97.8% vs. 97.6%, *p* = 0.999; 84.8% vs. 87.8%, *p* = 0.983) at 2 weeks after the second booster (**Figures 3B–D**; **Supplementary Table 1**). Despite no statistical significance, higher GMTs were observed after the second than after the first booster dose in IM compared with ID regimens and in mRNA-1273 compared with BNT162b2 vaccines (**Figure 3A**; **Supplementary Table 1**). In line with anti-RBD IgG GMCs, GMTs (95% CI) induced by the ID mRNA-1273 regimen were not significantly different from the IM BNT162b2 regimen at 2 weeks after the second booster against Wuhan (486.77 [321.48, 737.05] vs. 472.63 [291.24, 767.01], respectively; *p* = 0.072) (**Figure 3A**). For Omicron subvariants, a statistical difference was observed only for BA.1 (ID mRNA-1273 vs. IM BNT162b2: 323.97 [195.12, 537.91] vs. 542.82 [275.01, 1,071.40], *p* = 0.007) but not for BA.2 (328.28 [201.68, 534.33] vs. 472.96 [261.99, 853.81], *p* = 0.206) or BA.4/5 (205.10 [110.09, 382.11] vs. 308.27 [158.57, 599.29], *p* = 0.115) (**Figures 3B–D**). Both IM mRNA-1273 and BNT162b2 had higher GMTs than their respective ID regimens against BA.1 (*p* = 0.022 for mRNA-1273, *p* = 0.020 for BNT162b2), BA.2 (*p* = 0.039 for mRNA-1273, *p* = 0.433 for BNT162b2), and BA.4/5 (*p* = 0.608 for mRNA-1273, *p* = 0.031 for BNT162b2) subvariants at 2 weeks after the second booster (**Figures 3B–D**; **Supplementary Table 1**). Despite no statistical difference, GMTs were also higher across arms at 2 weeks after the second booster compared with at 2–4 weeks after the first booster (GMR [95% CI] of 1.34 [1.11, 1.62] for BA.2, *p* = 0.845; 1.10 [0.92, 1.33] for BA.4/5, *p* = 0.531), except for the BA.1 subvariant, which was statistically significant (1.71 [1.39, 2.11], *p* = 0.023) (**Figures 3B–D**; **Supplementary Table 1**). NAbs against Omicron subvariants were lower than those for Wuhan at 2 weeks after the second booster in all arms (GMT [95% CI]: 385.28 [295.36, 502.58], 429.52 [342.07, 539.33], and 209.88 [157.98, 278.83] for BA.1, BA.2, and BA.4/5 subvariants vs. 517.16 [426.33, 627.35] for Wuhan) (**Supplementary Table 1**).

### 3.4 Adverse events

**Figure 4:**
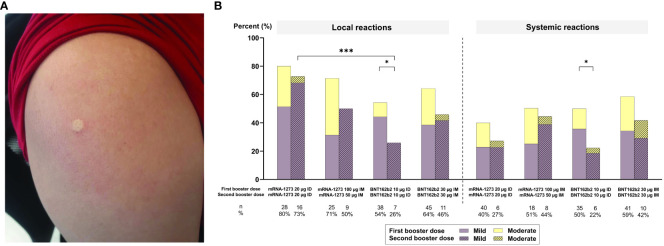
**(A)** An example of a wheal observed immediately after the administration of a second booster dose. **(B)** Local and systemic adverse events (AEs) reported 7 days after intramuscular (IM) or intradermal (ID) vaccination as a second booster. Only statistically significant *p*-values are displayed, with * and *** denoting *p* ≤ 0.05 and ≤ 0.001, respectively. µg, micrograms.

**Figure 4A** depicts wheal size immediately after ID vaccination. **Figure 4B** illustrates that both local and systemic AEs (mild and moderate) were less frequent after the second than the first booster, regardless of administrative route and vaccine type (*p* ≤ 0.05 for both local and systemic AEs for ID BNT162b2). Local AEs were significantly more frequent in ID mRNA-1273 arms than their respective BNT162b2 arms at 2 weeks after the second booster (*p* ≤ 0.001 for ID-mRNA-1273 vs. ID-BNT162b2; **Figure 4B**). This was also observed 2–4 weeks after the first booster. Systemic AEs were more frequent in IM vaccine regimens compared with their respective ID regimens at 2 weeks after the second booster (*p* = 0.304; **Figure 4B**; **Supplementary Table 2**). Systemic AEs also occurred more frequently in mRNA-1273 (27% for ID mRNA-1273 and 44% for IM mRNA-1273) compared with BNT162b2 (22% for ID BNT162b2 and 42% for IM BNT162b2) arms at 2 weeks after the second booster, regardless of administrative route (**Figure 4B**). The most common systemic AEs at 2 weeks after the second booster were myalgia (29.67%), headache (10.99%), and fatigue (10.99%) (**Supplementary Table 2**). There was no statistical difference in the frequency of these AEs across arms, regardless of dosing route or vaccine type (**Supplementary Figure 1**), except for fatigue, which was less prevalent 2 weeks after the second booster compared with 2–4 weeks after the first booster in those administered ID BNT162b2 (*p* = 0.05; **Supplementary Figure 1**). All local and systemic AEs were mild to moderate and resolved 2–3 days after administration of the second booster vaccines. No serious AEs were observed across arms.

## 4 Discussion

This was the first study to evaluate immunogenicity of repeated fractional ID mRNA COVID-19 vaccines as a second booster in a geriatric population and offer an alternative to standard IM regimens. Regardless of IM or ID route, we found that a second booster generated robust humoral immune responses that were similar or higher than those generated by a first booster against Wuhan and Omicron subvariants. While ID regimens generally induced lower humoral immune responses than IM regimens, ID-mRNA-1273 induced similar humoral immune responses to IM-BNT162b2. This suggests that fractional ID mRNA-1273 dosing regimens may provide similar protection to the standard IM BNT162b2 dosing regimens currently used globally to prevent severe COVID-19, with an additional advantage of lowering systemic AEs.

The increased anti-RBD IgG GMCs observed after a second booster, compared with baseline levels, noted in this study were consistent with previously reported values (12). Compared with adults aged ≥60 years old that received only a first booster, a second COVID-19 booster was associated with a 60%–70% vaccine effectiveness (VE) against hospitalization and >74% VE against death during the Omicron predominant period (5, 13–15). Consistent with a previous study, anamnestic antibody responses were observed after the administration of a second booster dose (16). This study also observed that NAb against Omicron subvariants were lower than Wuhan, and that mRNA-1273 vaccine regimens induced higher antibody responses than BNT162b2 vaccine regimens. A second booster of BNT162b2 or mRNA-1273 also induced higher NAb against Omicron subvariants compared with titers seen after a first booster. One crucial finding was that a higher proportion of individuals seroconverted against the Omicron subvariant after a second than after a first booster. This reflects the importance of a second booster against Omicron subvariant responses in adults aged ≥65 years. Further research is warranted, particularly against new Omicron subvariants (i.e., XBB, BQ.7).

This study’s findings build upon a previous study that found fractional ID vaccination of mRNA COVID-19 vaccines as a primary series and first booster were safe and immunogenic in adults (8). Despite the lower antibody responses observed in ID compared with IM regimens, similar T-cell responses were observed across groups at 2–4 weeks after a first booster (9). Whether higher ID vaccine dosage will improve immunogenicity remains to be determined.

Despite the tendency for ID regimens to have greater local AEs than IM regimens, a reduced frequency of systemic AEs was observed in this study after ID compared with IM arms of the same vaccine type. The frequency of both local and systemic AEs was lower at 2 weeks after the second booster compared with at 2–4 weeks after the first booster (9), regardless of administrative route and vaccine type. The lower observed AEs after the second compared with the first booster may promote vaccine uptake in geriatric populations. ID vaccination uses a fraction of the dose of standard IM vaccination, which also helps to alleviate vaccine supply issues. This is highly relevant for settings that have limited COVID-19 vaccine supplies, particularly those with next-generation COVID-19 vaccines.

This study had some limitations. First, the differences observed between vaccine routes and incidences of AEs should be interpreted with caution due to our small, predominantly female sample and non-randomized, unblinded study design. Second, we did not measure NAb at baseline (prior to administering the second booster). This may influence our ability to interpret the magnitude of peak antibody responses. However, based on RBD IgG data, NAb titers were expected to wane to similar levels across the groups. Third, we did not measure T-cell responses, and thus, could not evaluate improved cellular immunogenicity following the second booster. Finally, the technicalities of ID administration (i.e., requirements for specialized needles and techniques) may be less familiar than IM injection. Medical personnel of this study were trained to routinely administer ID rabies and BCG vaccines, and wheal size measurements were used to ensure proper techniques after injection (9).

In summary, we found that mRNA COVID-19 vaccines as a second booster with repeated fractional ID or IM routes generated robust humoral immune responses in a geriatric population. These humoral immune responses were greater after the second than after the first booster of the same vaccine regimen. Despite generating lower antibody responses than IM, ID vaccination offers an alternative vaccination strategy in settings with limited vaccine supplies and concerns regarding systemic AEs.

## Data availability statement

The original contributions presented in the study are included in the article/**Supplementary Material**. Further inquiries can be directed to the corresponding author.

## Ethics statement

The studies involving humans were approved by Siriraj Institutional Review Board (COA no. Si 335/2022). The studies were conducted in accordance with the local legislation and institutional requirements. The participants provided their written informed consent to participate in this study.

## Author contributions

PA: Conceptualization, Formal analysis, Methodology, Supervision, Writing – review & editing. SN: Conceptualization, Formal analysis, Methodology, Supervision, Writing – review & editing. MT: Data curation, Investigation, Resources, Writing – review & editing. NP: Data curation, Investigation, Resources, Writing – review & editing. WM: Data curation, Investigation, Resources, Writing – review & editing. ZT: Formal analysis, Writing – original draft, Writing – review & editing. PL: Formal analysis, Writing – review & editing. KC: Conceptualization, Formal analysis, Methodology, Supervision, Writing – original draft, Writing – review & editing.

## References

[1] Jo N Hidaka Y Kikuchi O Fukahori M Sawada T Aoki M. Impaired CD4+ T cell response in older adults is associated with reduced immunogenicity and reactogenicity of mRNA COVID-19 vaccination. Nat Aging (2023) 3(1):82–92. doi: 10.1038/s43587-022-00343-4 PMC10154196 37118516

[2] Cohen M Oster Y Moses A Spitzer A Benenson S. Israeli-Hospitals 4th Vaccine Working Group Association of receiving a fourth dose of the BNT162b vaccine with SARS-CoV-2 infection among health care workers in Israel. JAMA Netw Open (2022) 5:e2224657. doi: 10.1001/jamanetworkopen.2022.24657 35917125 PMC9346545

[3] Regev-Yochay G Gonen T Gilboa M Mandelboim M Indenbaum V Amit S. Efficacy of a fourth dose of Covid-19 mRNA vaccine against Omicron. New Engl J Med (2022) 386(14):1377–80. doi: 10.1056/NEJMc2202542 PMC9006792 35297591

[4] Munro AP Feng S Janani L Cornelius V Aley PK Babbage G. Safety, immunogenicity, and reactogenicity of BNT162b2 and mRNA-1273 COVID-19 vaccines given as fourth-dose boosters following two doses of ChAdOx1 nCoV-19 or BNT162b2 and a third dose of BNT162b2 (COV-BOOST): a multicentre, blinded, phase 2, randomised trial. Lancet Infect Diseases. (2022) 22(8):1131–41. doi: 10.1016/S1473-3099(22)00271-7 PMC9084623 35550261

[5] Arbel R Sergienko R Friger M Peretz A Beckenstein T Yaron S. Effectiveness of a second BNT162b2 booster vaccine against hospitalization and death from COVID-19 in adults aged over 60 years. Nat Med (2022) 28(7):1486–90. doi: 10.1038/s41591-022-01832-0 35468276

[6] Hickling J Jones R. Intradermal delivery of vaccines: A review of the literature and the potential for development for use in low- and middle income countries. Program Appropriate Technol Health (PATH) (2009) 27:1–94.

[7] Chatsiricharoenkul S Niyomnaitham S Posen HJ Toh ZQ Licciardi PV Wongprompitak P. Safety and immunogenicity of intradermal administration of fractional dose CoronaVac^®^, ChAdOx1 nCoV-19 and BNT162b2 as primary series vaccination. Front Immunol (2022) 13:1010835. doi: 10.3389/fimmu.2022.1010835 36268028 PMC9577032

[8] Niyomnaitham S Chatsiricharoenkul S Toh ZQ Senawong S Pheerapanyawaranun C Phumiamorn S. Evaluation of the safety and immunogenicity of fractional intradermal COVID-19 vaccines as a booster: A pilot study. Vaccines (2022) 10(9):1497. doi: 10.3390/vaccines10091497 36146575 PMC9505744

[9] Assantachai P Niyomnaitham S Chatthanawaree W Intalapaporn S Muangpaisan W Phannarus H. Immunogenicity and reactogenicity of mRNA COVID-19 vaccine booster administered by intradermal or intramuscular route in Thai Older adults. J Infect Dis (2023) 228(7):868–77. doi: 10.1093/infdis/jiad133 PMC10547455 37141388

[10] Koonpaew S Kaewborisuth C Srisutthisamphan K Wanitchang A Thaweerattanasinp T Saenboonrueng J. A single-cycle influenza a virus-based SARS-CoV-2 vaccine elicits potent immune responses in a mouse model. Vaccines (2021) 9(8):850. doi: 10.3390/vaccines9080850 34451975 PMC8402467

[11] Common Terminology Criteria for Adverse Events (CTCAE): U.S. Department of Health and Human Services (2017). Available at: https://ctep.cancer.gov/protocoldevelopment/electronic_applications/docs/CTCAE_v5_Quick_Reference_5x7.pdf.

[12] Canetti M Barda N Gilboa M Indenbaum V Mandelboim M Gonen T. Immunogenicity and efficacy of fourth BNT162b2 and mRNA1273 COVID-19 vaccine doses; three months follow-up. Nat Commun (2022) 13(1):7711. doi: 10.1038/s41467-022-35480-2 36513665 PMC9745767

[13] Magen O Waxman JG Makov-Assif M Vered R Dicker D Hernán MA. Fourth dose of BNT162b2 mRNA Covid-19 vaccine in a nationwide setting. New Engl J Med (2022) 386(17):1603–14. doi: 10.1056/NEJMoa2201688 PMC9020581 35417631

[14] Gazit S Saciuk Y Perez G Peretz A Pitzer VE Patalon T. Short term, relative effectiveness of four doses versus three doses of BNT162b2 vaccine in people aged 60 years and older in Israel: retrospective, test negative, case-control study. bmj (2022) 377:1–9. doi: 10.1136/bmj-2022-071113 PMC9127435 35609888

[15] Nordström P Ballin M Nordström A. Effectiveness of a fourth dose of mRNA COVID-19 vaccine against all-cause mortality in long-term care facility residents and in the oldest old: A nationwide, retrospective cohort study in Sweden. Lancet Regional Health-Europe. (2022) 21:100466. doi: 10.1016/j.lanepe.2022.100466 35855494 PMC9277096

[16] Canetti M Barda N Gilboa M Indenbaum V Asraf K Gonen T. Six-Month follow-up after a fourth BNT162b2 vaccine dose. New Engl J Med (2022) 387(22):2092–4. doi: 10.1056/NEJMc2211283 PMC9730934 36351266

