# Supplementary material for: Immunogenicity and reactogenicity of repeated intradermal mRNA COVID-19 vaccines administered as a second booster dose in a Thai geriatric population
Source: Front Immunol. 2024 Jan 11;14:1302041. doi: 10.3389/fimmu.2023.1302041 (PMC10810025; doi:10.3389/fimmu.2023.1302041)
Supplement: Supplementary file 1 [file DataSheet_1.docx]

Supplementary Material

# Supplementary Figures and Tables

## Supplementary Figure

## Supplementary Tables


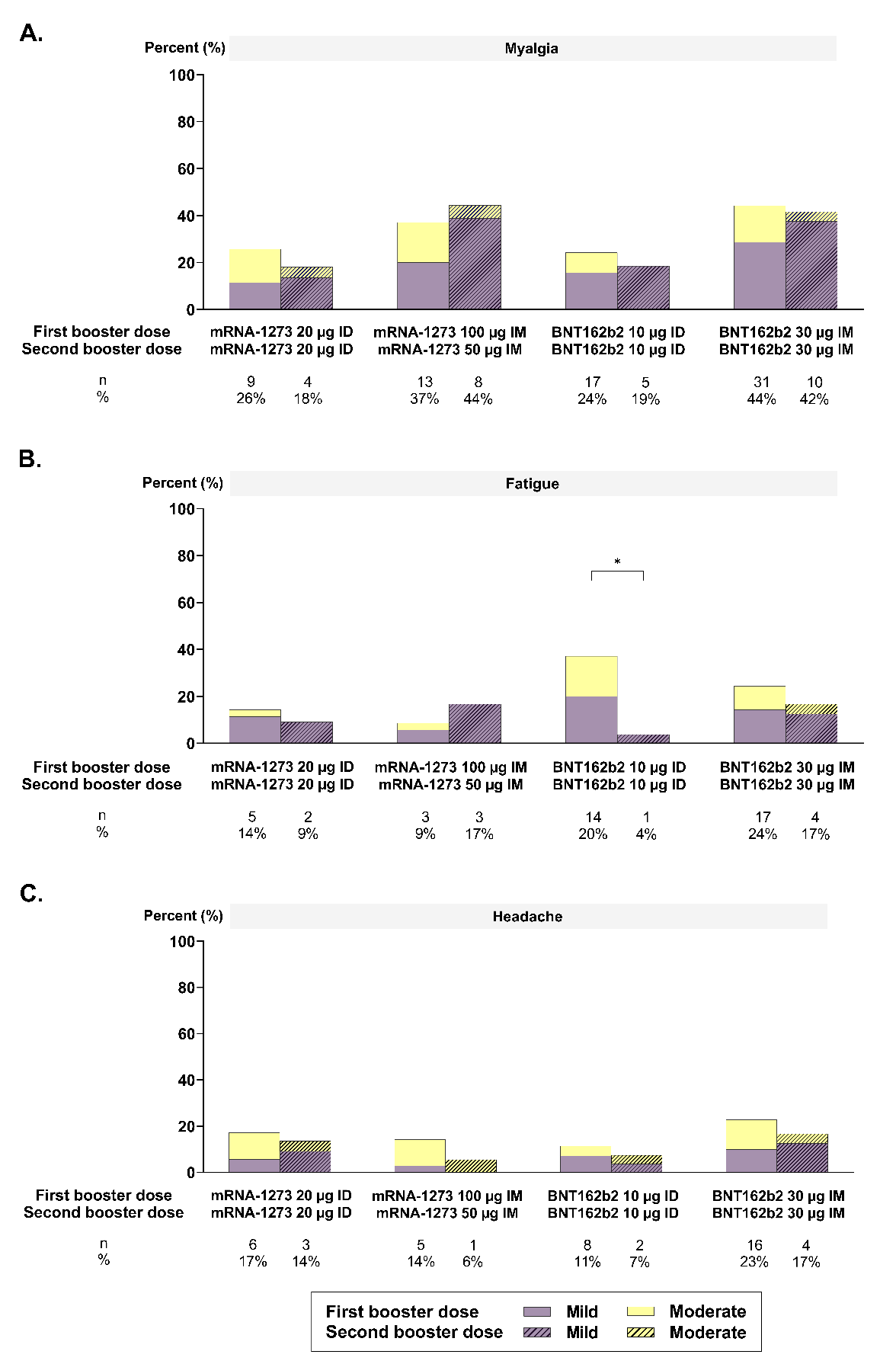


**Supplementary Figure 1.** Incidence of **(A)** myalgia, **(B)** fatigue, and **(C)** headache reported 7 days after intramuscular (IM) or intradermal (ID) vaccination as a fourth dose. Only statistically significant p-values are displayed, with * and *** denoting p ≤ 0.05 and ≤ 0.001, respectively. Abbreviations: µg, micrograms.

## Supplementary Tables

**Supplementary Table 1.** Immunogenicity of the first and second boosters by administration route and COVID-19 vaccine type.

|  | Vaccine Type | | | | | | | p-value |
| --- | --- | --- | --- | --- | --- | --- | --- | --- |
| First booster  -  Second booster | Total | mRNA-1273 20 µg ID  -  mRNA-1273 20 µg ID | | mRNA-1273 100 µg IM  -  mRNA-1273 50 µg IM | BNT162b2 10 µg ID  -  BNT162b2 10 µg ID | | BNT162b2 30 µg IM  -  BNT162b2 30 µg IM |  |
| Anti-RBD IgG levels (BAU/mL) | | | | | | | | |
| Number of subjects,  n (%) | 91  (100.00) | 22  (24.18) | | 18  (19.78) | 27  (29.67) | | 24  (26.37) |  |
| GMC before first booster  (95% CI) | 53.57  (41.51,  69.14) | 80.98  (48.20,  136.07) | | 44.39  (26.05,  75.62) | 43.02  (27.08,  68.35) | | 54.06  (29.87,  97.86) | 0.287 |
| GMC 2-4 weeks after first booster (95% CI) | 2,221.21  (1818.09, 2713.71) | 2,336.95  (1486.99,  3672.75) | | 3,561.10  (2335.35,  5429.93) | 1,483.27  (1097.78,  2004.11) | | 2,343.81  (1501.65,  3658.26) | 0.024* |
| GMC 16-17 weeks after first booster (95% CI) | 435.28  (336.53,  563.01) | 330.64  (191.98,  569.44) | | 681.74  (380.50,  1221.19) | 324.90  (215.18,  490.58) | | 555.80  (307.56,  1004.43) | 0.114 |
| Number of subjects,  n (%) | 87  (100.00) | 20  (22.99) | | 18  (20.69) | 26  (29.88) | | 23  (26.44) |  |
| GMC 2 weeks after second booster (95% CI) | 3,172.18  (2685.76, 3746.70) | 2,972.77  (2173.06,  4066.77) | | 5,320.38  (3725.03,  7598.98) | 2,341.98  (1867.83,  2936.50) | | 3,155.56  (2087.82,  4769.35) | 0.006* |
| Aggregate GMR: ID and IM 2 weeks after second booster (95% CI) | | 0.78  (0.67, 0.95) | | | 0.88  (0.72, 1.07) | | |  |
| p-value |  | 0.014* | | | 0.183 | | |  |
| GMR between 2 weeks after second booster and 2-4 weeks after first booster (95% CI) | 1.46  (1.26, 1.68) | 1.38  (0.97, 1.95) | | 1.49  (1.06, 2.10) | 1.57  (1.20, 2.07) | | 1.38  (1.05, 1.81) | 0.779 |
| GMR between 2-4 weeks and 16-17 weeks after the first booster (95% CI) | 5.10  (4.06, 6.41) | 7.07  (4.87, 10.26) | | 5.22  (2.68, 10.17) | 4.57  (2.95, 7.06) | | 4.22  (2.66, 6.69) | 0.404 |
| GMR between 2 weeks after second booster and 16-17 weeks after first booster (95% CI) | 7.26  (6.07, 8.70) | 10.00  (7.08, 14.13) | | 7.80  (4.75, 12.81) | 7.01  (5.34, 9.21) | | 5.41  (3.64, 8.06) | 0.064 |
| Pseudovirus Neutralization Titer (PVNT_50_) 2-4 weeks after first booster | | | | | | | | |
| Number of subjects,  n (%) | 91  (100.00) | 22  (24.18) | | 18  (19.78) | 27  (29.67) | | 24  (26.37) |  |
| GMT against Wuhan strain  2-4 weeks after first booster (95%CI) | 430.40  (343.05,  539.99) | 447.88  (254.48,  788.23) | | 695.79  (438.50,  1104.06) | 329.24  (244.22,  443.85) | | 391.26  (229.33,  667.55) | 0.147 |
| PVNT_50_ ≥ 1:40 against Wuhan strain 2-4 weeks after first booster, n (%) | 89  (97.80) | 22  (100.00) | | 18  (100.00) | 27  (100.00) | | 22  (91.67) | 0.161 |
| GMT against Omicron BA.1 strain 2-4 weeks after first booster (95%CI) | 225.56  (171.11,  297.33) | 293.33  (157.34,  546.85) | | 570.27  (333.76,  974.36) | 94.80  (61.08,  147.14) | | 234.44  (147.40,  372.87) | <0.001* |
| PVNT_50_ ≥ 1:40 against Omicron BA.1 2-4 weeks after first booster, n (%) | 78  (85.71) | 18  (81.82) | | 17  (94.45) | 21  (77.78) | | 22  (91.67) | 0.370 |
| GMT against Omicron BA.2 strain 2-4 weeks after first booster (95%CI) | 324.29  (254.18,  413.73) | 242.46  (137.02,  429.07) | | 498.84  (278.75,  892.70) | 241.98  (166.44,  351.79) | | 426.05  (257.95,  703.68) | 0.115 |
| PVNT_50_ ≥ 1:40 against Omicron BA.2 2-4 weeks after first booster, n (%) | 85  (93.41) | 19  (86.36) | | 17  (94.44) | 26  (96.30) | | 23  (95.83) | 0.621 |
| GMT against Omicron BA.4/5 strain 2-4 weeks after first booster (95%CI) | 207.22  (160.05,  268.29) | 183.48  (102.18,  329.47) | | 241.51  (136.28,  428.01) | 132.36  (90.63,  193.32) | | 253.81  (150.06,  429.29) | 0.044* |
| PVNT_50_ ≥ 1:40 against Omicron BA.4/5 strain 2-4 weeks after first booster, n (%) | 76  (83.52) | 18  (81.82) | | 16  (88.89) | 22  (81.48) | | 20  (83.33) | 0.954 |
| Pseudovirus Neutralization Titer (PVNT_50_) (2 weeks after second booster) | | | | | | | | |
| Number of subjects,  n (%) | 87  (100.00) | 20  (22.99) | | 18  (20.69) | 26  (29.88) | | 23  (26.44) |  |
| GMT against Wuhan strain 2 weeks after second booster (95%CI) | 517.16  (426.33,  627.35) | 486.77  (321.48,  737.05) | | 841.95  (563.65,  1257.66) | 418.71  (318.94,  549.70) | | 472.63  (291.24,  767.01) | 0.072 |
| PVNT_50_ ≥ 1:40 against Wuhan strain 2 weeks after second booster, n (%) | 86  (98.85) | 20  (100.00) | | 18  (100.00) | 26  (100.00) | | 22  (95.65) | 0.701 |
| Aggregate GMR: ID and IM against Wuhan strain 2 weeks after second booster (95%CI) |  | 0.79  (0.62, 1.01) | | | 0.95  (0.76, 1.91) | | |  |
| p-value |  | 0.055 | | | 0.644 | | |  |
| GMT against Omicron BA.1 strain 2 weeks after second booster (95%CI) | 385.28  (295.36, 502.58) | 323.97  (195.12,  537.91) | | 688.64  (459.37,  1032.35) | 217.45  (141.93,  333.17) | | 542.82  (275.01,  1071.40) | 0.007* |
| PVNT_50_ ≥ 1:40 against Omicron BA.1 2 weeks after second booster, n (%) | 83  (95.40) | 19  (95.00) | | 18  (100.00) | 25  (96.15) | | 21  (91.30) | 0.776 |
| Aggregate GMR: ID and IM against Omicron BA.1 strain 2 weeks after second booster (95%CI) | | 0.72  (0.55, 0.95) | | | 0.67  (0.48, 0.94) | | |  |
| p-value | | 0.022* | | | 0.020* | | |  |
| GMT against Omicron BA.2 strain 2 weeks after second booster (95%CI) | 429.52  (342.07, 539.33) | 328.28  (201.68,  534.33) | | 642.91  (415.36,  665.12) | 366.87  (260.10,  517.46) | | 472.96  (261.99,  853.81) | 0.206 |
| PVNT_50_ ≥ 1:40 against Omicron BA.2 2 weeks after second booster, n (%) | 85  (97.70) | 19  (95.00) | | 18  (100.00) | 26  (100.00) | | 22  (95.65) | 0.576 |
| Aggregate GMR: ID and IM against Omicron BA.2 strain 2 weeks after second booster (95%CI) | | 0.75  (0.57, 0.99) | | | 0.90  (0.68, 1.19) | | |  |
| p-value |  | 0.039* | | | 0.433 | | |  |
| GMT against Omicron BA.4/5 strain 2 weeks after second booster (95%CI) | 209.88  (157.98, 278.83) | 205.10  (110.09,  382.11) | | 255.46  (135.38,  482.03) | 132.70  (85.30,  206.44) | | 308.27  (158.57,  599.29) | 0.115 |
| PVNT_50_ ≥ 1:40 against Omicron BA.4/5 strain 2 weeks after second booster, n (%) | 75  (86.21) | 17  (85.00) | | 16  (88.89) | 22  (84.62) | | 20  (86.96) | 0.999 |
| Aggregate GMR: ID and IM against Omicron BA.4/5 strain 2 weeks after second booster (95%CI) | | 0.91  (0.63, 1.32) | | | 0.69  (0.50, 0.69) | | |  |
| p-value |  | 0.608 | | | 0.031* | | |  |
| GMR against Wuhan strain  between 2 weeks after second booster and 2-4 weeks after first booster | 0.53  (0.41, 0.69) | 0.41  (0.27, 0.62) | 0.40  (0.26, 0.63) | | 0.49  (0.33, 0.74) | 0.92  (0.46, 1.84) | | 0.071 |
| GMR against Omicron BA.1 strain between 2 weeks after second booster and 2-4 weeks after first booster | 1.71  (1.39, 2.11) | 1.17  (0.73, 1.86) | 1.21  (0.80, 1.82) | | 2.24  (1.57, 3.21) | 2.31  (1.46, 3.67) | | 0.023* |
| GMR against Omicron BA.2 strain between 2 weeks after second booster and 2-4 weeks after first booster | 1.34  (1.11, 1.62) | 1.38  (0.88, 2.15) | 1.29  (0.82, 2.02) | | 1.49  (1.08, 2.07) | 1.19  (0.80, 1.78) | | 0.845 |
| GMR against Omicron BA.4/5 strain between 2 weeks after second booster and 2-4 weeks after first booster | 1.10  (0.92, 1.33) | 1.09  (0.67, 1.79) | 1.06  (0.68, 1.64) | | 1.01  (0.74, 1.34) | 1.29  (0.89, 1.87) | | 0.531 |

Data are displayed as n (%) and Binding Antibody Units per mL (BAU/mL). Titers below the lower limit of detection (LLOD) of 1:40 were replaced with values of 20. Statistical significance is denoted by * (p ≤ 0.05). Abbreviations: µg, microgram; ID, intradermal; IM, intramuscular; GMC, geometric mean concentration; GMR, geometric mean ratio; GMT, geometric mean titer; CI, confidence interval.

**Supplementary Table 2.** Self-reported adverse events 0-7 days following the second booster dose.

|  | Vaccine Type | | | | | p-value |
| --- | --- | --- | --- | --- | --- | --- |
| First booster  -  Second booster | Total | mRNA-1273 20 µg ID  -  mRNA-1273 20 µg ID | mRNA-1273 100 µg IM  -  mRNA-1273 50 µg IM | BNT162b2 10 µg ID  -  BNT162b2 10 µg ID | BNT162b2 30 µg IM  -  BNT162b2 30 µg IM |  |
| Number of subjects, n (%) | 91 (100.00) | 22  (24.18) | 18  (19.78) | 27  (29.67) | 24  (26.37) |  |
| Local reactions, n (%)  Mild  Moderate | 43 (47.25)  41 (45.05)  2 (2.20) | 16 (72.73)  15 (68.18)  1 (4.55) | 9 (50.00)  9 (50.00)  0 (0.00) | 7 (25.93)  7 (25.93)  0 (0.00) | 11 (45.84)  10 (41.67)  1 (4.17) | 0.013* |
| Systemic reactions, n (%)  Mild  Moderate | 30 (32.96)  24 (26.37)  6 (6.59) | 6 (27.28)  5 (22.73)  1 (4.55) | 8 (44.44)  7 (38.89)  1 (5.56) | 6 (22.22)  5 (18.52)  1 (3.70) | 10 (41.67)  7 (29.17)  3 (12.50) | 0.304 |
| Myalgia, n (%)  Mild  Moderate | 27 (29.67)  24 (26.37)  3 (3.30) | 4 (18.19)  3 (13.64)  1 (4.55) | 8 (44.45)  7 (38.89)  1 (5.56) | 5 (18.52)  5 (18.52)  0 (0.00) | 10 (41.67)  9 (37.50)  1 (4.17) | 0.128 |
| Fatigue, n (%)  Mild  Moderate | 10 (10.99)  9 (9.89)  1 (1.10) | 2 (9.09)  2 (9.09)  0 (0.00) | 3 (16.67)  3 (16.67)  0 (0.00) | 1 (3.70)  1 (3.70)  0 (0.00) | 4 (16.67)  3 (12.50)  1 (4.17) | 0.403 |
| Headache, n (%)  Mild  Moderate | 10 (10.99)  6 (6.59)  4 (4.40) | 3 (13.64)  2 (9.09)  1 (4.55) | 1 (5.56)  0 (0.00)  1 (5.56) | 2 (7.40)  1 (3.70)  1 (3.70) | 4 (16.67)  3 (12.50)  1 (4.17) | 0.627 |
| Fever, n (%)  Mild  Moderate | 1 (1.10)  0 (0.00)  1 (1.10) | 0 (0.00)  0 (0.00)  0 (0.00) | 1 (5.56)  0 (0.00)  1 (5.56) | 0 (0.00)  0 (0.00)  0 (0.00) | 0 (0.00)  0 (0.00)  0 (0.00) | 0.198 |
| Vomiting, n (%)  Mild  Moderate | 2 (2.20)  2 (2.20)  0 (0.00) | 0 (0.00)  0 (0.00)  0 (0.00) | 1 (1.56)  1 (1.56)  0 (0.00) | 0 (0.00)  0 (0.00)  0 (0.00) | 1 (4.17)  1 (4.17)  0 (0.00) | 0.449 |
| Diarrhea, n (%)  Mild  Moderate | 2 (2.20)  2 (2.20)  0 (0.00) | 1 (4.55)  1 (4.55)  0 (0.00) | 0 (0.00)  0 (0.00)  0 (0.00) | 0 (0.00)  0 (0.00)  0 (0.00) | 1 (4.17)  1 (4.17)  0 (0.00) | 0.697 |

Data are displayed as n (%). Statistical significance is denoted by * (p ≤ 0.05). Abbreviations: µg, microgram; ID, intradermal; IM, intramuscular.
